# Fire acupuncture for anti-LGI1 antibody autoimmune encephalitis: a case report

**DOI:** 10.3389/fnins.2023.1203915

**Published:** 2023-07-19

**Authors:** Yu Liu, Yu Gong, Xiao-li Wu, Xiao-min Hao, Ji-peng Liu, Yin-yin Li, Ke-zhen Yang, Xin-yu Gao, Jing Zhang, Lin Zhang, Xu-dong Zhang, Jun Wang, Qing-guo Liu

**Affiliations:** ^1^Dongzhimen Hospital, Beijing University of Chinese Medicine, Beijing, China; ^2^School of Acupuncture-Moxibustion and Tuina, Beijing University of Chinese Medicine, Beijing, China; ^3^Department of Chinese Medicine, Beijing Jishuitan Hospital, Beijing, China

**Keywords:** fire acupuncture, autoimmune diseases, cognitive dysfunction, glucocorticoids, inflammation

## Abstract

Autoimmune encephalitis, a class of encephalitis, is clinically characterized by multifocal or diffuse brain injury, including aberrant mental behavior, convulsions, and near-event memory impairment. In this article, we describe a female patient with autoimmune encephalitis who tested positive for leucine-rich glioma inactivated 1 (LGI1) antibodies and had hippocampal inflammatory edema in the lesion area. During the first 3 months of her illness, the patient primarily experienced memory loss, the onset of rigid twitching in her extremities that lasted for 1 min while in remission, and incontinence. After gamma globulin administration, methylprednisolone shock, and other symptomatic therapies during hospitalization, the patient’s psychiatric symptoms and seizures improved considerably; however, she did not fully recover her memory. After receiving fire acupuncture for 6 months, the patient’s understanding, orientation, and calculation skills improved considerably. Her memory and mental state were also improved at the follow-up visit. In this case, the use of fire acupuncture for the treatment of autoimmune encephalitis resulted in favorable outcomes with important benefits for conditions affecting the central nervous system; however, more convincing data are required to support the effectiveness of this treatment method.

## 1. Introduction

Autoimmune encephalitis is a novel inflammatory disease of the central nervous system mediated by antibodies directed against neurotransmitter receptors or neuronal surface proteins. The clinical syndrome is complex, and its manifestations vary depending on the type of antibody involved; however, it mainly presents with acute or subacute onset of cognitive impairment, epileptic seizures, psychobehavioral abnormalities, and a wide variety of movement disorders (Dutra et al., 2018).

Anti-leucine-rich glioma inactivated 1 (LGI1) antibody-positive encephalitis accounts for approximately 30% of limbic encephalitis-associated antibodies and is typically characterized by faciobrachial dystonic seizures, cognitive decline, hyponatremia, anti-LGI1 antibody-positive serum or cerebrospinal fluid, and abnormal magnetic resonance imaging (MRI) signals in the medial temporal lobe or hippocampus (Linnoila et al., 2014; Plantone, 2018).

The treatment regimens for autoimmune encephalitis are based on the treatment principles for other life-threatening autoimmune diseases. Various therapies, including corticosteroids, intravenous immunoglobulin, plasma exchange, rituximab, and cyclophosphamide, are currently in use (Newman et al., 2016). However, there are no specific treatment options for certain antibody-mediated types of encephalitis.

## 2. Case description

The treatment of autoimmune encephalitis with acupuncture has not yet been documented. Here, we report a case of autoimmune encephalitis (anti-LGI1 antibody encephalitis) treated with fire needles.

**Figure 1:**
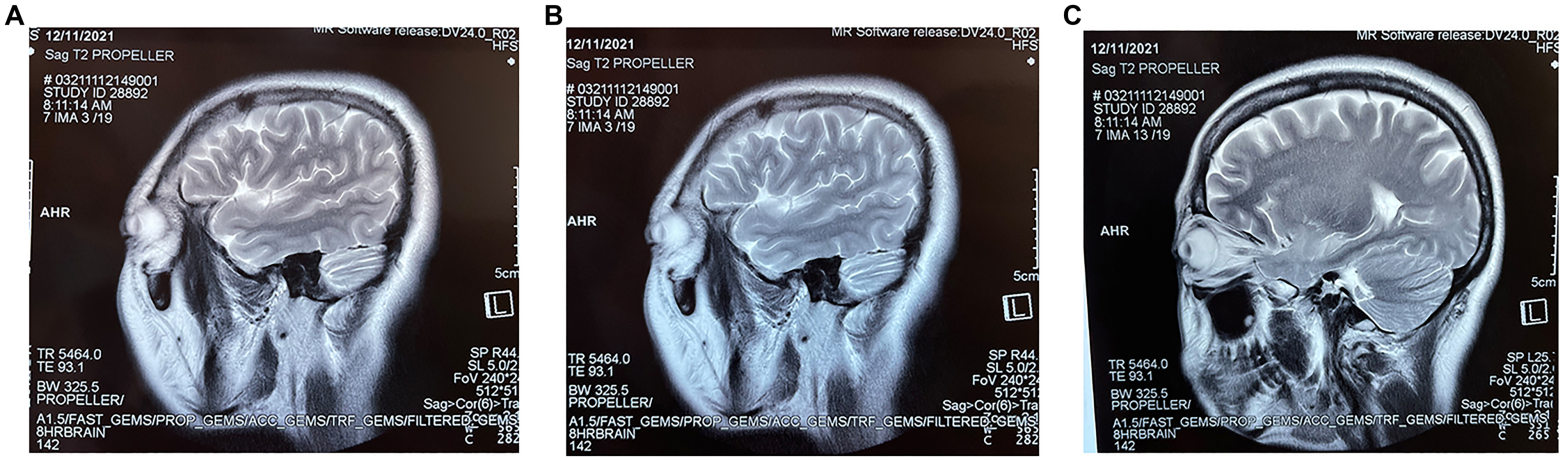
**(A–C)** are three different sections of the sagittal position of the first MRI at the patient’s admission.

On November 12, 2021, a 44-year-old female patient was admitted to the hospital with memory loss for 3 months and convulsions for 2 days. She presented with symptoms such as limb stiffness, incontinence, and consciousness impairment that lasted for 1 min and then resolved. Positron electron tomography showed swelling of the left hippocampus with slightly decreased density and increased glucose metabolism, cranial MRI showed abnormal signals in the bilateral anterior temporal lobes with left medial temporal lobe swelling (Figure 1), and blood-biochemical tests showed a blood sodium concentration of 106 mmol/L. The EEG showed fully guided short-to-long-range diffuse 2.0–4.5 Hz slow-wave activity of medium-high amplitudes with high amplitudes during interictal, awake, and sleep periods and higher amplitudes in the frontal and temporal regions. Anti-LGI1 antibodies were present in serum and lumbar puncture cerebrospinal fluid. Based on the patient’s clinical presentation, LGI1 autoimmune encephalitis was diagnosed. The patient was treated with 20 g gamma globulin intravenously for 5 days, combined with methylprednisolone shock therapy (methylprednisolone sodium succinate 1,000 mg/d intravenously for 3 days, then 500 mg/d intravenously for 3 days, after which the dose was reduced to 40–80 mg/d intravenously for 2 weeks), along with levetiracetam as an antiepileptic drug, pantoprazole sodium enteric solution tablets for gastric protection, and potassium chloride extended-release tablets for potassium supplementation. The patient was discharged after 20 days of hospitalization with substantial symptom improvement. After discharge, glucocorticoid treatment was further tapered (60 mg of prednisone acetate once a day, 5 mg every 2 weeks until it was stopped). Although the disease was controlled in the acute phase, the patient reported distant and recent memory losses, resulting in loss of the patient’s ability to perform daily activities, sleep with dreaminess and easy awakening, rapid weight gain after taking hormonal drugs, menstrual disorders, dry eyes, and depressed mood. Although these symptoms can be improved by oral administration of sleeping pills, antidepressants, progestins, and other drugs, these treatment options can only alleviate the symptoms but do not address the root cause of the problem. As the patient turned down these options, she visited a traditional Chinese medicine (TCM) clinic on December 23, 2021.

**Figure 2:**
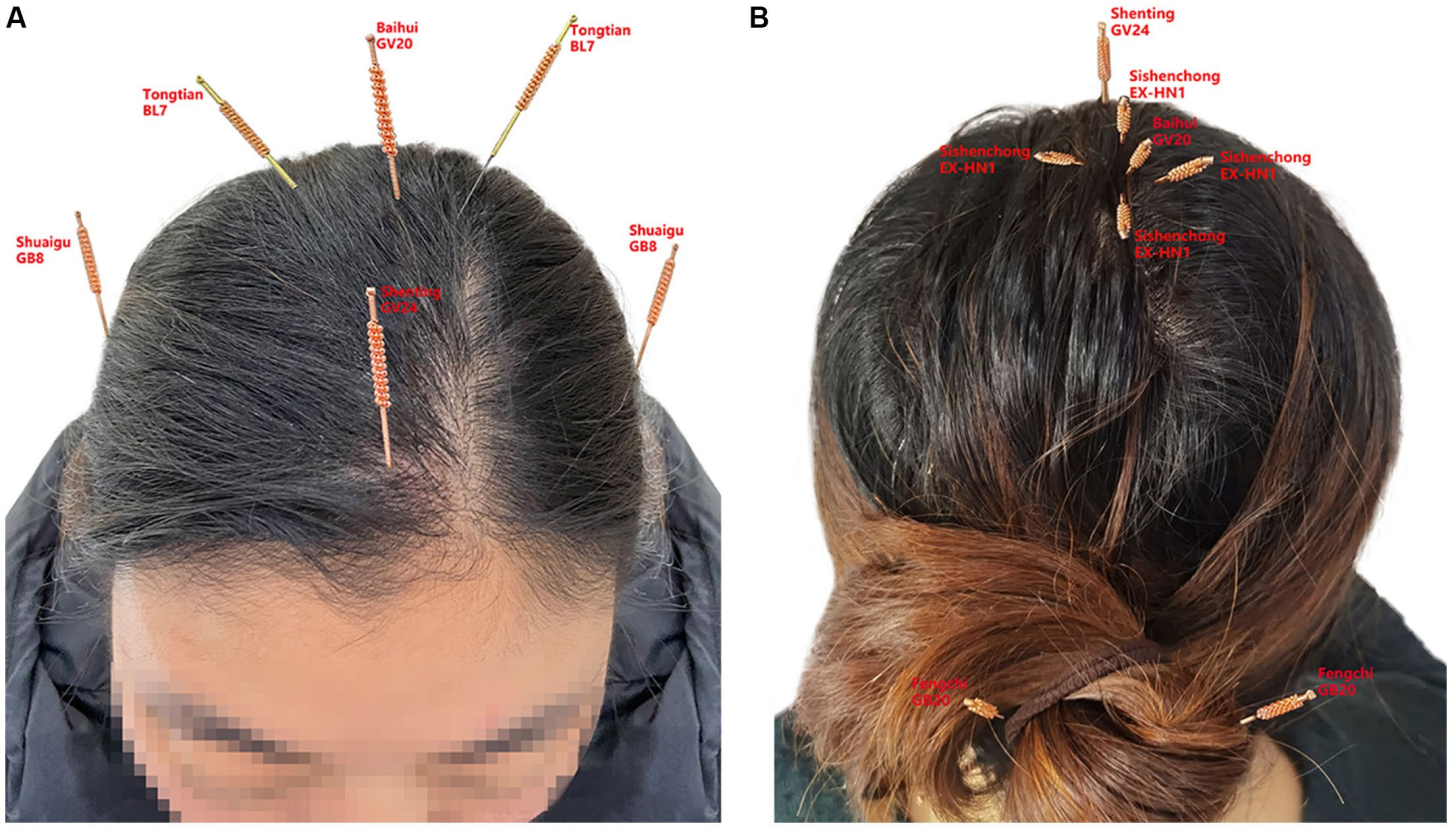
**(A)** represents the front of the head, **(B)** represents the back of the head, and the small labels inside represent the acupuncture points of the head, Baihui (GV20), Shenting (GV24), Sishen cong (EX-HN1), Tongtian (BL7), Shuaigu (GB8), and Fengchi (GB20).

The acupuncturist, with more than 30 years of experience, arranged for her to receive fire acupuncture treatment while continuing to take prednisone. During the treatment, a 0.4 × 25 mm fine fire needle was selected and held in the left hand with an alcohol lamp, while the right hand held the needle in a pen grip, with the tip and part of the needle body inserted into the flame. The acupuncture points were selected according to the functional brain regions classified by Western medicine, with projection points of the prefrontal, temporal, parietal, and occipital regions of the head on the scalp. For example, the Baihui (GV20), Sishencong (EX-HN1), and Tongtian (BL7) points of the parietal lobe, the Shenting (GV24) point of the prefrontal lobe, the Shuaigu (GB8) point of the temporal lobe, and the Fengchi (GB20) point of the occipital lobe were selected (Figure 2). The operator must be strictly disinfected before the operation of the fire needle; operation process to “red” “accurate” “fast,” the operation site after the needle prick mild itching or small red swelling, hand scratching is strictly prohibited, keep needle hole clean, dry, so as to avoid infection of the needle hole, if there is redness, swelling, heat and pain and other inflammatory reactions, available fire needle local puncture or oral anti-inflammatory drugs. Treatment with fire needles is prohibited for patients with blood clotting disorders, and patients with diabetes mellitus should use fire needles with caution (Huang et al., 2013). After treatment, the head was kept warm and observed for half an hour in the consultation room before the patient left the clinic. She underwent acupuncture treatment twice a week, and the same acupuncture point was not repeatedly used within a week; instead, the treatment points were adjusted according to the patient’s condition in a timely manner.

**Figure 3:**
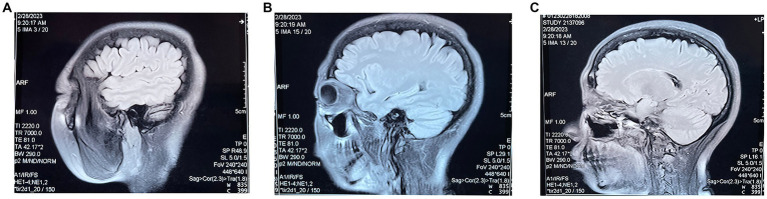
**(A–C)** are three different sections of the sagittal position of the MRI re-examined 10 months after the patient’s fire needle treatment.

In fact, although the patient was out of life-threatening conditions during her hospitalization and no longer suffered from loss of consciousness, grand mal seizures, and incontinence, the patient’s distant and recent memory was severely lost and her calculation, orientation, and comprehension were severely affected when she came to the TCM clinic, so she was not considered to have entered the clinical recovery period, but rather the clinical treatment period. During the first 3 months of treatment with fire acupuncture, the patient reported a gradual recovery of recent memory and an improvement in calculation and comprehension. The EEG of the hospital also showed that “during the interictal period, a small amount of slow wave activity intermittently appeared in the left frontal and temporal areas during sleep, with a medium amplitude of 1.9–3.0 HZ, compared with the EEG at the beginning of the attack, the slow wave activity was significantly reduced,” and entered the clinical recovery period. The patient continued the fire acupuncture treatment for another 2 months, and all of the patient’s cognitive functions recovered significantly, and the patient’s weight increased due to taking hormone drugs for several months, and her emotional impatience and menstrual disorder also gradually returned to normal. After complete discontinuation of hormones, another month of fire acupuncture treatment alone, the patient’s condition was more stable than before, no abnormalities, reaching a stable stage of clinical recovery, and follow-up observation was recommended. To date, the patient has been followed up for 10 months without recurrence. Cranial MRI was reviewed for abnormal signaling of both anterior temporal lobes with a slight swelling of the medial left temporal lobe (Figure 3). Repeat EEG showed no obvious electrical abnormalities during the interictal period.

## 3. Discussion

In this case, conventional hormonal medications did not significantly improve the patient’s memory. After six courses of acupuncture treatment, memory recovery was successfully achieved, and the quality of life was also improved considerably by the effective alleviation of the side effects of hormonal treatment and maintenance of disease remission after the withdrawal of late hormonal medications, suggesting the effectiveness of acupuncture for the treatment of autoimmune encephalitis.

LGI1 is a secreted protein that is mainly distributed in the hippocampus and temporal cortex. It is expressed in neuronal axons and at the initiation of glutamatergic synapses (Hivert et al., 2019). LGI1 is involved in brain development, neuronal excitation, and synaptic transmission. In addition, some types of anti-LGI1 antibody encephalitis cause hippocampal atrophy and microstructural damage (Finke et al., 2017; Szots et al., 2017), which may be partly due to complement-dependent cytotoxic effects and consequential reduction in the number of neurons (Bien et al., 2012; Bauer and Bien, 2016). Recent studies have shown that monoclonal antibodies against LGI1 increase cellular excitability and glutamatergic synaptic transmission velocity in hippocampal CA3 neurons, which may contribute to the pathogenicity of LGI1 antibodies (Kornau et al., 2020; Ramberger et al., 2020).

Previous studies have demonstrated that the hippocampal CA1 region is associated with cognitive function and contains various neurons important for information processing. The CA3 area is rich in glutamate receptors, specifically NMDA receptors, which are critical for the formation of associative memories (Doron et al., 2022). In addition, they play an important role in encoding new spatial information in short-term memory (Rolls, 2022). Memory, cognitive abilities, and emotion regulation are all regulated by the hippocampus, a key region of the limbic system (Zeidman and Maguire, 2016). The patient in this case had left hippocampal swelling, resulting in severe memory loss and cognitive dysfunction.

Based on replicable randomized controlled trials and functional fMRI studies, the understanding of acupuncture has been shifted to a less mysterious and more quantifiable science. MRI studies have demonstrated how the stimulation of certain acupoints results in the activation of corresponding brain areas (Zeidman and Maguire, 2016). The exact mechanism of action of acupuncture is not fully understood. However, it is thought to stimulate the central nervous system by releasing specific neurotransmitters and hormones. It has been hypothesized that the specific pathways, neurotransmitters, and hormones involved differ in different symptoms and disease states (Cai and Shen, 2018; Guo and Ma, 2019; Yu et al., 2020). Based on the TCM theory, both conventional and fire acupuncture play a therapeutic role by regulating the balance of qi and blood in the body, and as a whole, help relieve and cure diseases. The amount of stimulation of the body during the fire acupuncture process is much greater than that of milli-needle; secondly, the patient’s attention during the fire acupuncture operation is much greater than that of milli-needle; again, the intensity of excitation foci formed in the cerebral cortex by fire acupuncture is much greater than that of milli-needle stimulation. Fire acupuncture has a dual role of acupuncture therapy and moxibustion therapy, not only providing the stimulating effect of the needles but also the stimulating effect of the heat. Through warm stimulation of the acupuncture points, fire acupuncture aims to warm the meridians and regulate the function of internal organs (Yue et al., 2019). Modern research has found that fire acupuncture can improve blood circulation and increase metabolism. Infrared thermograms of fire acupuncture sites demonstrated that the temperature of lesions treated by fire acupuncture increased significantly, suggesting improved local blood circulation and metabolism (Xing et al., 2019). Fire acupuncture can eliminate local tissue congestion, edema, exudation, and adhesions, improve local tissue calcification, ischemia, contracture, and other pathological changes, promote lesion liquefaction and necrosis, stimulate the benign regulatory mechanisms of the body, and enhance immune function (Wan et al., 2022). Previous studies have shown that fire acupuncture significantly increases brain-derived neurotrophic factor expression, promotes endogenous neural stem cell proliferation and differentiation into neurons, inhibits neuronal apoptosis, reduces inflammation through autophagy, and promotes the recovery of motor neuron function (Qiu et al., 2021).

Head fire acupuncture is a modern acupuncture technique that combines the acupuncture methods of Chinese medicine and the functions of the brain areas, using needles to stimulate different locations (points, zones, or areas) of the scalp. The effects of head stimulation on cerebral cortex function may be achieved by stimulating specific anatomical structures. Stimulation of the skin, fascia, muscles, and periosteum of the head can activate the functional areas of the cerebral cortex through the midbrain, thalamus, and brainstem, and the influence of different anatomical structures on brain function is regular and variable (Kim et al., 2020). Therefore, during treatment with fire needles, the angle and depth of acupuncture should be adjusted according to the patient’s reaction under the needle to achieve better treatment results.

Several studies suggest that acupuncture stimulation increases cyclic-AMP response binding protein activity and reduces neuronal cell loss in the hippocampal region (Yun et al., 2017; Xie et al., 2021), which can improve memory impairment in various disorders, including Alzheimer’s disease, Parkinson’s disease, and stroke (Wattanathorn and Sutalangka, 2014; Page et al., 2021). It has also been demonstrated that acupuncture is beneficial for post-stroke rehabilitation, including aphasia, insomnia, neuroplasticity, depression, and cognitive impairment in diseases including Parkinson’s disease, spinal cord injury, and stroke (Dou et al., 2016; Widrin, 2018; Xiao et al., 2018; Xiong et al., 2020; Li et al., 2021). Previous studies have revealed that acupuncture reorganizes motor-related networks, including the primary motor network, sensorimotor network, default mode network, and language-related brain areas, including the frontal, temporal, parietal, and occipital lobes of the inferior frontal gyrus, and cognition-related brain regions (Li S. et al., 2017; Li Y. et al., 2017; Lee et al., 2019; Zhang et al., 2021). In addition, some studies have shown that acupuncture can stimulate bilateral regions, regulate the whole-brain network, and enhance functional connections. This suggests that acupuncture can specifically regulate bilateral homeostasis in the brain and the entire brain network and functional connections (Li et al., 2015).

Acupuncture also has positive effects on autoimmune diseases. It regulates immunity by stimulating the central nervous system and peripheral neuroendocrine immune mechanisms. Neuromediators, hormones, cytokines, and other substances that constitute the continuum have a broad regulatory spectrum of immune activity to safeguard all known immunopathological responses, including a range of pathological links that contribute to autoimmune diseases (Dantzer, 2018). Moreover, with the help of the peripheral components of the neuroendocrine-immune system, the pathological links of the main etiology of the disease can be successfully regulated (Wang et al., 2020).

Previous studies have also shown that acupuncture regulates immunity by stimulating natural killer cell activity, regulating Th1/Th2 balance, reducing apoptosis, and increasing lymphokine-induced killer cytotoxic activity. Acupuncture also regulates the biochemical synergy between electroacupuncture and neurotrophic factors and the mobilization of corticosterone, endorphins, and adrenocorticotropic hormone. Acupuncture has both pro-and anti-inflammatory effects and improves the function of all immune cells. Increased release of endogenous opioid peptides is a crucial step in the activation of the immune system that can be achieved through acupuncture (Cabioğlu and Cetin, 2008; Takahashi et al., 2009; Kim and Bae, 2010; Torres-Rosas et al., 2014). Although there is no clear record of acupuncture treatment for autoimmune encephalitis, acupuncture has a large number of evidence-based reports for autoimmune diseases, such as autoimmune encephalomyelitis, multiple sclerosis, rheumatoid arthritis, and Guillain-Barré syndrome (Lee et al., 2016; Criado et al., 2017; Xu et al., 2018; Li et al., 2022).

Previous studies have shown that acupuncture improves neurological and autoimmune diseases. However, medical records and randomized controlled trials on acupuncture for autoimmune encephalitis are lacking. TCM focuses on the overall concept, and acupuncture treatment is based on the patient’s inquisition and dialectical therapy; however, from the perspective of evidence-based medicine, acupuncture has enough evidence to support its effectiveness in the treatment of autoimmune and central nervous system diseases.

In this case report, we combined acupuncture with the brain function division of Western medicine to select acupuncture points on the patient’s head for fire acupuncture treatment to improve symptoms. The Baihui acupoint (GV20) is located at the top of the head and is a common acupuncture point for relieving dizziness, headache, and anxiety, due to the regulatory effect of acupuncture GV20 on the endocrine system, immune system, and nervous system (Deng et al., 2016). Baihui (GV20) and Shenting (GV24) are often used as acupoint combinations to treat nervous system diseases (Zhan et al., 2016; Li S. et al., 2017; Li Y. et al., 2017). The Sishencong is located 1 inch on the left and right sides of the Baihui point. It has a calming and strengthening effect on the mind and plays a major role in promoting sleep, nourishing the spirit, and enhancing memory.

Mild focal seizures and faciobrachial dystonic seizures in anti-LGI1 antibody encephalitis mostly precede memory impairment seizures. Later in the disease course, 63% of patients have tonic-clonic seizures. The initial MRI shows high T74 signals in the hippocampus of 2% of all patients. These lesions regularly evolve into medial temporal lobe sclerosis (44%). However, 80% of patients have a significant response to immunotherapy, leading to early seizure response and slow cognitive recovery (van Sonderen et al., 2016). Although the incidence of anti-LGI1 antibody encephalitis is low and can be controlled by early clinical diagnosis and treatment, its prognosis is not objective, with memory impairment and spatial disorientation (van Sonderen et al., 2017). The recurrence rate of anti-LGI1 antibody encephalitis is 16.2%, and the median time between the first onset and first recurrence is 5 months (Qiao et al., 2021). At more than 2 years of follow-up, the majority of surviving patients report still having mild cognitive impairment, and 86% of patients are affected by persistent amnesia during their disease course. Relapses are common, occurring even 8 years after the initial disease. The two-year morbidity and mortality rates are 19% (van Sonderen et al., 2016). Therefore, timely follow-up after discontinuation of long-term immunotherapy is required to prevent disease progression or recurrence. Western medicine has only a few effective measures for preventing disease recurrence, and symptomatic treatment is an advantage of Western medicine. We performed fire acupuncture treatment for another 1 month after the patient discontinued hormonal drugs and followed up for 1 year, and the patient’s condition considerably improved. Acupuncture may have improved this patient’s condition; however, more cases and randomized controlled trials are needed to confirm that acupuncture can prevent or reduce recurrence, which is also consistent with the concept of Chinese medicine, “not treating one’s own disease before it occurs.”

## 4. Conclusion

Our case demonstrates that scalp fire acupuncture can improve memory and cognitive function in autoimmune encephalitis, attenuate the side effects of hormonal drugs, improve patients’ quality of life, and, most importantly, reduce disease recurrence. Scalp fire needling may be an effective, cost-effective, and safe adjuvant treatment; however, further studies with larger sample sizes are needed to investigate its mechanism and efficacy.

## Data availability statement

The raw data supporting the conclusions of this article will be made available by the authors, without undue reservation.

## Ethics statement

Written informed consent was obtained from the individual(s) for the publication of any potentially identifiable images or data included in this article.

## Author contributions

YL contributed to the conception and design of the study and drafted the manuscript. YG contributed to drafting the text and preparing figures. X-mH, X-lW, J-pL, K-zY, and Y-YL translated, revised, and proofread the manuscript. X-yG, JZ, and LZ collected case data. X-dZ, JW, and Q-gL participated in the acupuncture intervention. All authors contributed to the article and approved the submitted version.

## Funding

This study was funded by a grant from the National Natural Science Foundation of China (grant no. 82074553).

## Conflict of interest

The authors declare that the research was conducted in the absence of any commercial or financial relationships that could be construed as a potential conflict of interest.

## Publisher’s note

All claims expressed in this article are solely those of the authors and do not necessarily represent those of their affiliated organizations, or those of the publisher, the editors and the reviewers. Any product that may be evaluated in this article, or claim that may be made by its manufacturer, is not guaranteed or endorsed by the publisher.

## Abbreviations

LGI1: Leucine-rich glioma inactivated 1
MRI: Magnetic resonance imaging
TCM: Traditional Chinese medicine

## References

[ref1] Bauer J. Bien C. G. (2016). Neuropathology of autoimmune encephalitides. Handb. Clin. Neurol. 133, 107–120. doi: 10.1016/B978-0-444-63432-0.00007-4 27112674

[ref2] Bien C. G. Vincent A. Barnett M. H. Becker A. J. Blümcke I. Graus F.. (2012). Immunopathology of autoantibody-associated encephalitides: clues for pathogenesis. Brain 135, 1622–1638. doi: 10.1093/brain/aws082, PMID: 22539258

[ref3] Cabioğlu M. T. Cetin B. E. (2008). Acupuncture and immunomodulation. Am. J. Chin. Med. 36, 25–36. doi: 10.1142/S0192415X08005552 18306447

[ref4] Cai W. Shen W. D. (2018). Anti-apoptotic mechanisms of acupuncture in neurological diseases: a review. Am. J. Chin. Med. 46, 515–535. doi: 10.1142/S0192415X1850026X 29595076

[ref5] Criado M. B. Santos M. J. Machado J. Gonçalves A. M. Greten H. J. (2017). Effects of acupuncture on gait of patients with multiple sclerosis. J. Altern. Complement. Med. 23, 852–857. doi: 10.1089/acm.2016.0355, PMID: 28410453

[ref6] Dantzer R. (2018). Neuroimmune interactions: From the brain to the immune system and vice versa. Physiol. Rev. 98, 477–504. doi: 10.1152/physrev.00039.2016, PMID: 29351513 PMC5866360

[ref7] Deng D. Liao H. Duan G. Liu Y. He Q. Liu H.. (2016). Modulation of the default mode network in first-episode, drug-naïve major depressive disorder via acupuncture at Baihui (GV20) acupoint. Front. Hum. Neurosci. 10:230. doi: 10.3389/fnhum.2016.00230, PMID: 27242492 PMC4869560

[ref8] Doron A. Rubin A. Benmelech-Chovav A. Benaim N. Carmi T. Refaeli R.. (2022). Hippocampal astrocytes encode reward location. Nature 609, 772–778. doi: 10.1038/s41586-022-05146-6, PMID: 36045289

[ref9] Dou J.-M. Huang C.-Y. Ge Y.-J. Ji Y.-Y. Jiang Z.-D. Wan Z.-Y.. (2016). Analysis of the clinical value of modified scalp acupuncture in rehabilitation treatment of stroke. World Latest Inf. 57:175. doi: 10.3969/j.issn.1671-3141.2016.57.139

[ref10] Dutra L. A. Abrantes F. Toso F. F. Pedroso J. L. Barsottini O. G. P. Hoftberger R. (2018). Autoimmune encephalitis: a review of diagnosis and treatment. Arq. Neuro Psiquiatr. 76, 41–49. doi: 10.1590/0004-282X20170176 29364393

[ref11] Finke C. Prüss H. Heine J. Reuter S. Kopp U. A. Wegner F.. (2017). Evaluation of cognitive deficits and structural hippocampal damage in encephalitis with leucine-rich, glioma-inactivated 1 antibodies. JAMA Neurol. 74, 50–59. doi: 10.1001/jamaneurol.2016.4226, PMID: 27893017

[ref12] Guo X. Ma T. (2019). Effects of acupuncture on neurological disease in clinical-and animal-based research. Front. Integr. Neurosci. 13:47. doi: 10.3389/fnint.2019.00047, PMID: 31543763 PMC6729102

[ref14] Hivert B. Marien L. Agbam K. N. Faivre-Sarrailh C. (2019). ADAM22 and ADAM23 modulate the targeting of the Kv1 channel associated protein LGI1 to the axon initial segment. J. Cell Sci. 132:jcs219774. doi: 10.1242/jcs.219774, PMID: 30598502

[ref15] Huang C. J. Huang Y. J. Chen C. Y. (2013). The developmental origin of fire acupuncture therapy. Zhongguo Zhen Jiu 33, 455–458. Chinese. doi: 10.13703/j.0255-2930.2013.05.022 PMID: 23885626

[ref16] Kim S. K. Bae H. (2010). Acupuncture and immune modulation. Auton. Neurosci. 157, 38–41. doi: 10.1016/j.autneu.2010.03.010 20399151

[ref17] Kim H. Mawla I. Lee J. Gerber J. Walker K. Kim J.. (2020). Reduced tactile acuity in chronic low back pain is linked with structural neuroplasticity in primary somatosensory cortex and is modulated by acupuncture therapy. NeuroImage 217:116899. doi: 10.1016/j.neuroimage.2020.116899, PMID: 32380138 PMC7395964

[ref18] Kornau H. C. Kreye J. Stumpf A. Fukata Y. Parthier D. Sammons R. P.. (2020). Human cerebrospinal fluid monoclonal LGI1 autoantibodies increase neuronal excitability. Ann. Neurol. 87, 405–418. doi: 10.1002/ana.25666, PMID: 31900946

[ref19] Lee M. J. Jang M. Choi J. Lee G. Min H. J. Chung W. S.. (2016). Bee venom acupuncture alleviates experimental autoimmune encephalomyelitis by upregulating regulatory T cells and suppressing Th1 and Th17 responses. Mol. Neurobiol. 53, 1419–1445. doi: 10.1007/s12035-014-9012-2, PMID: 25579380

[ref20] Lee J. H. Kyeong S. Kang H. Kim D. H. (2019). Structural and functional connectivity correlates with motor impairment in chronic supratentorial stroke: a multimodal magnetic resonance imaging study. Neuroreport 30, 526–531. doi: 10.1097/WNR.0000000000001247, PMID: 30932970

[ref21] Li N. Guo Y. Gong Y. Zhang Y. Fan W. Yao K.. (2021). The anti-inflammatory actions and mechanisms of acupuncture from acupoint to target organs via neuro-immune regulation. J. Inflam. Res. 14, 7191–7224. doi: 10.2147/JIR.S341581, PMID: 34992414 PMC8710088

[ref22] Li M. K. Li Y. J. Zhang G. F. Chen J. Q. Zhang J. P. Qi J.. (2015). Acupuncture for ischemic stroke: cerebellar activation may be a central mechanism following Deqi. Neural Regen. Res. 10, 1997–2003. doi: 10.4103/1673-5374.172318, PMID: 26889189 PMC4730825

[ref23] Li S. Tan J. Zhang H. Huang G. Deng D. Jiang Q. (2017). Discussion on rules of acupoint selection for vascular dementia. Zhongguo Zhen Jiu 37, 785–790. Chinese. doi: 10.13703/j.0255-2930.2017.07.026 29231557

[ref24] Li Y. Wang Y. Liao C. Huang W. Wu P. (2017). Longitudinal brain functional connectivity changes of the cortical motor-related network in subcortical stroke patients with acupuncture treatment. Neural Plast. 2017:5816263. doi: 10.1155/2017/5816263, PMID: 29375914 PMC5742470

[ref25] Li J. Xu D. Liu Y. Cao Y. He J. Liao M. (2022). Acupuncture treatment of Guillain-Barré syndrome after using immune checkpoint inhibitors: A case report. Front. Neurol. 13:908282. doi: 10.3389/fneur.2022.908282, PMID: 35720101 PMC9201402

[ref26] Linnoila J. J. Rosenfeld M. R. Dalmau J. (2014). Neuronal surface antibody-mediated autoimmune encephalitis. Semin. Neurol. 34, 458–466. doi: 10.1055/s-0034-1390394, PMID: 25369441 PMC4838036

[ref27] Newman M. P. Blum S. Wong R. C. Scott J. G. Prain K. Wilson R. J.. (2016). Autoimmune encephalitis. Intern. Med. J. 46, 148–157. doi: 10.1111/imj.12974 26899887

[ref28] Page M. J. McKenzie J. E. Bossuyt P. M. Boutron I. Hoffmann T. C. Mulrow C. D.. (2021). The PRISMA 2020 statement: an updated guideline for reporting systematic reviews. BMJ 372:n71. doi: 10.1136/bmj.n71, PMID: 33782057 PMC8005924

[ref29] Plantone D. (2018). Striatum involvement in LGI1 limbic encephalitis. Clin. Psychopharmacol. Neurosci. 16, 508–509. doi: 10.9758/cpn.2018.16.4.508, PMID: 30466226 PMC6245290

[ref30] Qiao S. Wu H. K. Liu L. L. Wang M. L. Zhang R. R. Han T.. (2021). Clinical features and long-term outcomes of anti-leucine-rich glioma-inactivated 1 encephalitis: a multi-center study. Neuropsychiatr. Dis. Treat. 17, 203–212. doi: 10.2147/NDT.S292343, PMID: 33531809 PMC7846830

[ref31] Qiu X. Gao Y. Zhang Z. Cheng S. Zhang S. (2021). Fire acupuncture versus conventional acupuncture to treat spasticity after stroke: A systematic review and meta-analysis. PLoS One 16:e0249313. doi: 10.1152/physrev.00039.2016, PMID: 33836008 PMC8034732

[ref32] Ramberger M. Berretta A. Tan J. M. M. Sun B. Michael S. Yeo T.. (2020). Distinctive binding properties of human monoclonal LGI1 autoantibodies determine pathogenic mechanisms. Brain 143, 1731–1745. doi: 10.1093/brain/awaa104, PMID: 32437528 PMC7296845

[ref33] Rolls E. T. (2022). The hippocampus, ventromedial prefrontal cortex, and episodic and semantic memory. Prog. Neurobiol. 217:102334. doi: 10.1016/j.pneurobio.2022.102334, PMID: 35870682

[ref34] Szots M. Blaabjerg M. Orsi G. Iversen P. Kondziella D. Madsen C. G.. (2017). Global brain atrophy and metabolic dysfunction in LGI1 encephalitis: a prospective multimodal MRI study. J. Neurol. Sci. 376, 159–165. doi: 10.1016/j.jns.2017.03.020, PMID: 28431605

[ref35] Takahashi T. Sumino H. Kanda T. Yamaguchi N. (2009). Acupuncture modifies immune cells. J. Exp. Clin. Med. 1, 17–22. doi: 10.1016/S1878-3317(09)60006-1

[ref36] Torres-Rosas R. Yehia G. Peña G. Mishra P. del Rocio Thompson-Bonilla M. Moreno-Eutimio M. A.. (2014). Dopamine mediates vagal modulation of the immune system by electroacupuncture. Nat. Med. 20, 291–295. doi: 10.1038/nm.3479, PMID: 24562381 PMC3949155

[ref37] van Sonderen A. Roelen D. L. Stoop J. A. Verdijk R. M. Haasnoot G. W. Thijs R. D.. (2017). Anti-LGI1 encephalitis is strongly associated with HLA-DR7 and HLA-DRB4. Ann. Neurol. 81, 193–198. doi: 10.1002/ana.24858, PMID: 28026046

[ref38] van Sonderen A. Thijs R. D. Coenders E. C. Jiskoot L. C. Sanchez E. de Bruijn M. A.. (2016). Anti-LGI1 encephalitis: Clinical syndrome and long-term follow-up. Neurology 87, 1449–1456. doi: 10.1212/WNL.0000000000003173 27590293

[ref39] Wan R. Fan Y. Zhao A. Xing Y. Huang X. Zhou L.. (2022). Comparison of efficacy of acupuncture-related therapy in the treatment of rheumatoid arthritis: A network meta-analysis of randomized controlled trials. Front. Immunol. 13:829409. doi: 10.3389/fimmu.2022.829409, PMID: 35320944 PMC8936080

[ref40] Wang Y. F. Chen X. Sha L. Kendrick K. M. Lee L. T. O. Cheng C. H. K. (2020). Editorial: Neuroendocrine research in health and disease. Front. Neurosci. 14:176. doi: 10.3389/fnins.2020.00176, PMID: 32265620 PMC7105775

[ref41] Wattanathorn J. Sutalangka C. (2014). Laser acupuncture at HT7 acupoint improves cognitive deficit, neuronal loss, oxidative stress, and functions of cholinergic and dopaminergic systems in animal model of Parkinson's disease. Evid. Based Complement. Alternat. Med. 2014:937601. doi: 10.1155/2014/937601, PMID: 25161693 PMC4138813

[ref42] Widrin C. (2018). Scalp acupuncture for the treatment of motor function in acute spinal cord injury: a case report. J. Acupunct. Meridian Stud. 11, 74–76. doi: 10.1016/j.jams.2018.01.002, PMID: 29436375

[ref43] Xiao L. Y. Wang X. R. Yang Y. Yang J. W. Cao Y. Ma S. M.. (2018). Applications of acupuncture therapy in modulating plasticity of central nervous system. Neuromodulation 21, 762–776. doi: 10.1111/ner.12724, PMID: 29111577

[ref44] Xie L. Liu Y. Zhang N. Li C. Sandhu A. F. Williams G. 3rd. (2021). Electroacupuncture improves M2 microglia polarization and glia anti-inflammation of hippocampus in Alzheimer's disease. Front. Neurosci. 15:689629. doi: 10.3389/fnins.2021.689629, PMID: 34646113 PMC8502881

[ref45] Xing M. Yan X. Sun X. Wang S. Zhou M. Zhu B.. (2019). Fire needle therapy for moderate-severe acne: A PRISMA systematic review and meta-analysis of randomized controlled trials. Complement. Ther. Med. 44, 253–260. doi: 10.1016/j.ctim.2019.04.009, PMID: 31126563

[ref46] Xiong J. Zhang Z. Ma Y. Li Z. Zhou F. Qiao N.. (2020). The effect of combined scalp acupuncture and cognitive training in patients with stroke on cognitive and motor functions. NeuroRehabilitation 46, 75–82. doi: 10.3233/NRE-192942, PMID: 32039871

[ref47] Xu Y. Hong S. H. Zhao X. Wang S. Xu Z. Ding S.. (2018). Acupuncture alleviates rheumatoid arthritis by immune-network modulation. Am. J. Chin. Med. 46, 997–1019. doi: 10.1142/S0192415X18500520, PMID: 30001644

[ref48] Yu C. C. Du Y. J. Wang S. Q. Liu L. B. Shen F. Wang L.. (2020). Experimental evidence of the benefits of acupuncture for Alzheimer's disease: an updated review. Front. Neurosci. 14:549772. doi: 10.3389/fnins.2020.549772, PMID: 33408601 PMC7779610

[ref49] Yue X. Y. Feng Z. Q. Yu X. Y. Hu J. M. He X. J. Shu S. (2019). Fire-needle acupuncture for upper limb spastic paralysis after stroke: Study protocol for a randomized controlled trial. J Integr Med. 17, 167–172. doi: 10.1016/j.joim.2019.03.002, PMID: 30922849

[ref50] Yun Y. C. Jang D. Yoon S. B. Kim D. Choi D. H. Kwon O. S.. (2017). Laser acupuncture exerts neuroprotective effects via regulation of Creb, Bdnf, Bcl-2, and Bax gene expressions in the hippocampus. Evid. Based Complement. Alternat. Med. 2017:7181637. doi: 10.1155/2017/7181637, PMID: 28408940 PMC5376935

[ref51] Zeidman P. Maguire E. A. (2016). Anterior hippocampus: the anatomy of perception, imagination and episodic memory. Nat. Rev. Neurosci. 17, 173–182. doi: 10.1038/nrn.2015.24, PMID: 26865022 PMC5358751

[ref52] Zhan J. Pan R. Guo Y. Zhan L. He M. Wang Q.. (2016). Acupuncture at Baihui (GV 20) and Shenting (GV 24) combined with basic treatment and regular rehabilitation for post-stroke cognitive impairment: a randomized controlled trial. Zhongguo Zhen Jiu 36, 803–806. Chinese. doi: 10.13703/j.0255-2930.2016.08.007 29231563

[ref53] Zhang J. Lu C. Wu X. Nie D. Yu H. (2021). Neuroplasticity of acupuncture for stroke: an evidence-based review of MRI. Neural Plast. 2021:2662585. doi: 10.1155/2021/2662585, PMID: 34456996 PMC8397547

